# Design strategies of polyvinyl alcohol-based composites for EMI shielding: from matrix modulation to structural engineering

**DOI:** 10.1039/d6ra05816g

**Published:** 2026-08-17

**Authors:** Rui Zheng, Jiang Yang, Zihuan Weng, Yuzhu Xiong

**Affiliations:** a Northern Alabama International College of Engineering and Technology, Guizhou University Guiyang 550025 China; b Department of Polymer Materials and Engineering, College of Materials and Metallurgy, Guizhou University Guiyang 550025 China xyzhu789@126.com

## Abstract

Polyvinyl alcohol (PVA) is widely used as the polymer matrix in electromagnetic interference (EMI) shielding composites due to its excellent film-forming properties, abundant hydroxyl active sites and good biocompatibility. However, PVA's intrinsic hydrophilicity makes it prone to water absorption and swelling under high-humidity conditions, leading to the destabilisation of the conductive network. Furthermore, conductive nanofillers such as MXene and graphene are prone to agglomeration, whilst high-conductivity inorganic fillers can cause interfacial impedance mismatch, resulting in significant electromagnetic wave reflection losses, which limit the improvement of the overall performance of shielding materials. This paper provides a systematic review of the influence of PVA synthesis processes and its molecular chain structure on the microstructure and electrical properties of composite systems. It focuses on elucidating the latest research findings on three major categories of PVA-based shielding materials—films, aerogels/foams and hydrogels—and analyses the mechanisms of synergy between different conductive fillers and the PVA matrix, as well as the patterns of electromagnetic loss in different structures. Finally, the paper outlines future research directions for the design and engineering applications of PVA in high-performance absorptive electromagnetic shielding composites.

## Abstract 2

This review summarizes the synthesis and multi-morphology (films, aerogels, and hydrogels) fabrication of PVA-based composites, with a focus on their electromagnetic shielding mechanisms, and reveals the structure–shielding performance correlation.

## Graphical abstract

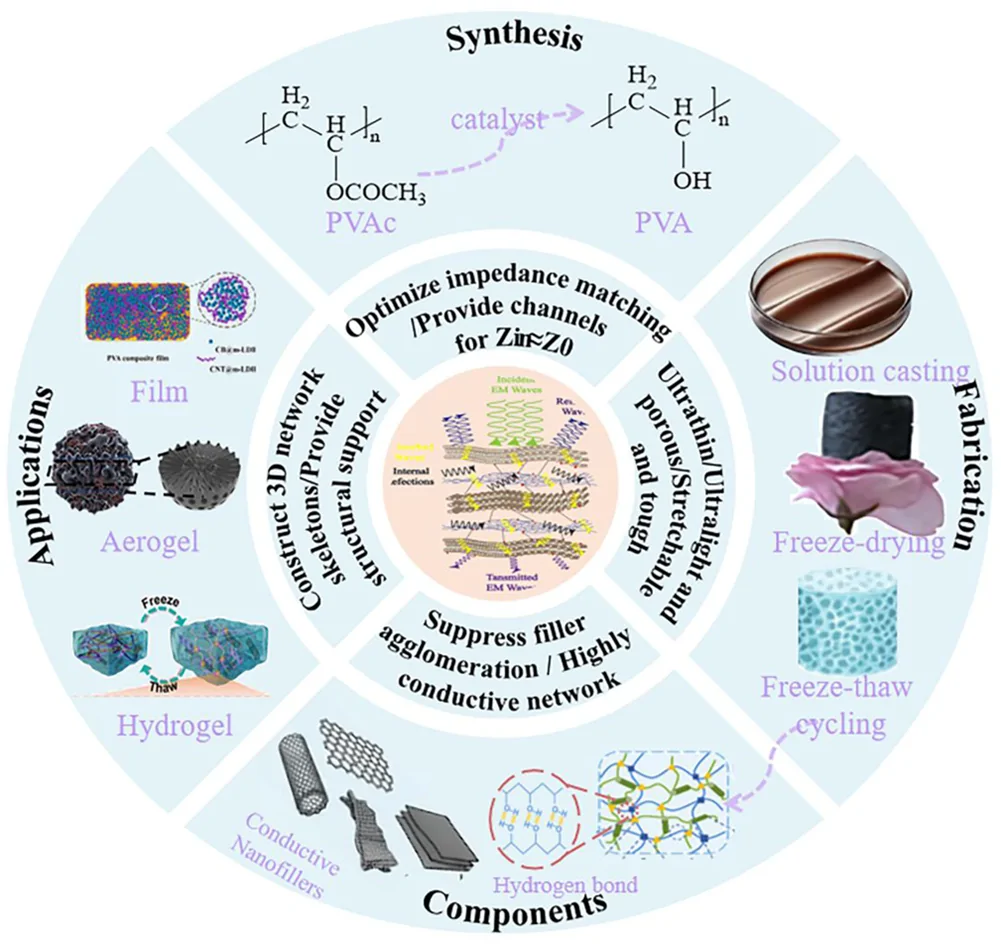

## 1 Introduction

As a vital carrier of information, electromagnetic waves have profoundly transformed the way of life in human society.^1,2^ Today, electromagnetic wave technology has deeply penetrated every aspect of human life, forming an application system that spans multiple fields including communications, healthcare, defence, industry and smart homes.^3–5^ In the civilian sector, technologies such as 5G and Wi-Fi have broken down spatial barriers, making electromagnetic waves an indispensable part of daily life.^6,7^ In the medical field, equipment and technologies such as magnetic resonance imaging (MRI) utilise electromagnetic waves to provide precise support for the diagnosis and treatment of diseases.^8^ In the military sector, equipment such as radar relies on electromagnetic waves and has become one of the key competitive advantages in modern warfare.^9,10^

Although the widespread use of electromagnetic waves has brought us convenience, exposure to large amounts of electromagnetic waves in the air can cause severe electromagnetic radiation pollution.^11^ Despite their convenience, the ubiquity of electromagnetic waves in the environment has led to severe electromagnetic radiation pollution, which threatens the normal operation of electronic equipment and poses potential risks to human health.^12^

In recent years, novel conductive nanomaterials, represented by MXene, graphene and carbon nanotubes, have demonstrated excellent shielding potential. However, these nanofillers are highly prone to agglomeration in practical applications, whilst single inorganic materials often suffer from high mechanical brittleness and difficulties in maintaining structural stability at the macroscopic scale. Against this backdrop, polyvinyl alcohol (PVA), as a polymer matrix, has demonstrated unique and irreplaceable advantages, as well as far-reaching research significance: firstly, the PVA molecular chains are rich in hydroxyl functional groups, which can form strong hydrogen-bond networks with terminal groups on the surfaces of materials such as MXene or with carbon nanomaterials, effectively suppressing the agglomeration of highly reactive nanofillers and constructing a uniform, continuous network of conductivity and polarisation loss. Secondly, PVA's excellent water solubility and ease of processing enable it to adapt readily to a variety of forming processes (such as solution casting and freeze-drying), thereby allowing the free design of cross-scale structures—ranging from two-dimensional ultrathin films to three-dimensional complex biomimetic porous scaffolds—at both the macroscopic and microscopic scales. Thirdly, the incorporation of PVA compensates for the mechanical shortcomings of inorganic shielding materials, endowing the composites with ultra-high elongation at break, excellent toughness and even self-healing capabilities, thereby meeting the requirements for mechanical stability in complex operating environments.

In light of this, this paper systematically reviews research progress on PVA-based composites in the field of electromagnetic shielding. Focusing on impedance matching and multidimensional structure formation, it investigates the influence of PVA molecular chain characteristics (such as hydrogen-bond networks, crystallisation behaviour and cross-linking patterns) on the complex permittivity and loss factor of the composite system. Although many recent reviews have summarised major advances in the field of electromagnetic shielding, they are often limited by their specific focus. For example, reviews on general polymer-based EMI shielding materials^13–15^ typically provide a broad overview of the interactions between the matrix and fillers, but lack an in-depth analysis of specific molecular chain modifications. Similarly, reviews focusing on MXene and carbon-based shielding materials^16–18^ primarily emphasise the inherent high electrical conductivity and two-dimensional structural advantages of the fillers, whilst neglecting the topological and chemical modulation provided by the polymer matrix. Although some recent literature has systematically reviewed hydrogel-based EMI materials, emphasising the role of three-dimensional networks and water molecules,^19–21^ these studies are limited to wet-state systems and do not cover dry-state macrostructures such as aerogels or dense films. Furthermore, although there is a wealth of existing reviews on PVA composites, these have primarily focused on traditional applications in the biomedical, packaging or conventional flexible electronics sectors, with a lack of systematic discussion regarding their electromagnetic energy dissipation mechanisms.^22,23^

To address these gaps, the unique contribution of this review lies in its explicit focus on the physicochemical properties of PVA molecular chains, treating them as a functional dielectric modifier rather than merely an inert binder. In contrast to existing reviews, this paper systematically compares the structure–property relationships and electromagnetic loss mechanisms of three distinct macroscopic morphologies (two-dimensional films, three-dimensional aerogels/foams, and water-rich hydrogels). Through a comprehensive analysis of the synergistic interactions between various conductive fillers and the PVA matrix, this review reveals how PVA actively regulates impedance matching through ‘dielectric dilution’ and the modulation of bound water.^24–26^ This not only aligns with the current trend in electromagnetic protection materials, which is shifting from ‘reflective’ to ‘highly absorptive’ types,^27^ but also provides a novel model and approach for addressing increasingly complex electromagnetic radiation pollution and developing environmentally friendly, high-performance protective electronic devices.

## 2 Preparation and processing of PVA-based composites

### 2.1 Chemical synthesis and molecular structure regulation of PVA

Polyvinyl alcohol (PVA) is typically produced from polyvinyl acetate (PVAc) *via* an alcoholysis reaction (hydrolysis or saponification), as shown in Fig. 1. By controlling the alcoholysis conditions, the degree of alcoholysis and the degree of polymerisation of PVA can be adjusted, thereby yielding PVA products with different properties. In recent years, researchers have introduced numerous innovations and optimisations to PVA synthesis methods, with the aim of achieving more precise control over the molecular structure of PVA. Köhler^28^ and colleagues investigated the processing properties of thermoplastic polyvinyl alcohol *via* melt spinning, demonstrating PVA's potential for application in the field of fibre precursors. At the level of molecular structure design, researchers have achieved systematic control over the stereoregularity of PVA through the stereoselective controlled radical polymerisation of vinylboron monomers and side-chain substitution reactions, opening up new avenues for the precise regulation of PVA's sequence structure.^29^ With regard to molecular weight control, Arman^30^ and colleagues employed cobalt-mediated radical polymerisation (CMRP) for the controlled polymerisation of vinyl acetate, successfully preparing PVA with tunable molecular weight. This effectively addressed the issues of poor printability and excessively rapid degradation rates associated with conventional PVA in biomedical applications. This strategy, by precisely controlling the chain length of PVAc and thereby regulating the molecular weight of PVA following alcoholysis, has laid the foundation for the preparation of PVA materials with designed properties. From the perspective of electromagnetic shielding, the precise regulation of PVA's molecular weight and stereoregularity directly determines its rheological behaviour and crystallinity when compounded with conductive fillers, thereby having a profound impact on the continuity of the charge transport network in the final composite material. An in-depth examination of the molecular chain structure of PVA reveals that its crystallinity, which is determined by its stereoregularity, directly governs, from a microscopic topological perspective, the hopping conduction and electron tunnelling effects of conductive fillers within the matrix.^31^ Studies have shown that the introduction of carbon nanofillers significantly affects the crystallisation behaviour of the PVA matrix—XRD characterisation indicates that the doping of MWCNTs and GNPs increases the crystallinity of PVA from approximately 35% to approximately 40%,^32^ and that hydrogen bonding between f-MWCNTs and PVA chains also promotes the ordered arrangement of PVA chains.^33^

**Fig. 1:**
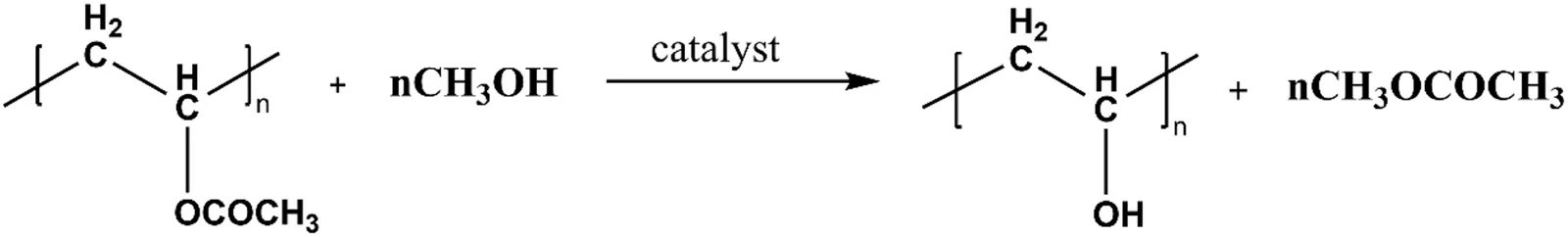
Schematic diagram of the alcoholysis of polyvinyl acetate to form PVA.

On the one hand, the crystalline regions of PVA are intrinsically insulating and densely packed; within composite systems, they exert a significant ‘volume exclusion effect’ on conductive nanofillers (such as MXene and carbon nanotubes).^34,35^ Based on this theoretical framework, Amrin and Deshpande directly verified the existence and applicability of this effect in the PVA/MWCNT-COOH system using the volume exclusion theory and the Garboczi model.^36^ That is, the fillers are unable to penetrate the PVA crystalline regions and are forced to aggregate within the amorphous bands of the non-crystalline regions. This microscopic phase separation behaviour enables the fillers to rapidly interlock at extremely low macroscopic loading levels, exceeding the percolation threshold,^37^ thereby constructing highly efficient electron transport pathways at low cost. On the other hand, excessive crystallinity leads to an increase in the size of the insulating crystalline regions, which inadvertently widens the insulating barrier between the conductive frameworks in the amorphous regions.^38^ According to the quantum tunnelling effect,^36^ when the width of the insulating barrier exceeds the electron hopping limit (typically a few nanometres), the probability of electron transmission decays exponentially, thereby compromising macroscopic electrical conductivity. Consequently, balancing the repulsive volume effect of the PVA insulating crystalline regions with the tunnelling energy barrier constitutes the fundamental physical mechanism for fine-tuning the complex permittivity and achieving high shielding performance.

It is worth exploring in greater depth that there are significant differences in how conductive fillers with varying dimensions and surface chemical properties modulate the crystallisation behaviour of PVA. From a geometric and topological perspective, one-dimensional carbon nanotubes (CNTs) and silver nanowires (AgNWs) primarily rely on their aspect ratio to form physical entanglements and volume exclusion effects within the PVA matrix, thereby enriching themselves in the amorphous regions and indirectly inducing chain segment orientation.^32,34^ In contrast, two-dimensional MXene nanosheets possess a larger specific surface area and stronger interfacial coupling capabilities; their surfaces, rich in oxygen-containing/fluorine-containing functional groups (such as –OH and –F) on their surfaces can form a dense network of hydrogen bonds with the hydroxyl groups of PVA.^39–41^ This not only promotes heterogeneous nucleation of PVA on the MXene surface but, due to strong interfacial bonding, significantly restricts the large-scale motion of polymer chains when the filler content exceeds a certain critical value, thereby inhibiting the complete growth of crystals.^42^ Consequently, differences in critical crystallinity are essentially the result of a trade-off between the topological dimensions of the filler and the strength of interfacial chemical bonding.^43^ Furthermore, when the filler content approaches the conductive leakage threshold, the formation of the conductive network reaches a dynamic equilibrium with the restricted motion of the polymer chains,^44^ at which point even minute changes in crystallinity exert a decisive influence on the width of the insulating barrier (*i.e.* the size of the crystalline regions), thereby exerting a non-linear regulatory effect on the macroscopic electrical conductivity and dielectric loss characteristics of the composite system *via* the quantum tunnelling effect.^36,38^

### 2.2 Processing techniques for PVA-based composites

In the face of the increasingly severe problem of electromagnetic radiation pollution, electromagnetic shielding technology has emerged. The core essence of electromagnetic shielding lies in utilising shielding materials to block, attenuate and dissipate electromagnetic waves. For PVA-based composites, the microstructural architecture and the ultimate electromagnetic wave dissipation pathways depend heavily on the processing techniques employed. As shown in Fig. 2, three core preparation methods—solution casting, freeze-drying and freeze–thaw cycling—can be used to engineer distinctly different microstructures and shielding mechanisms.

**Fig. 2:**
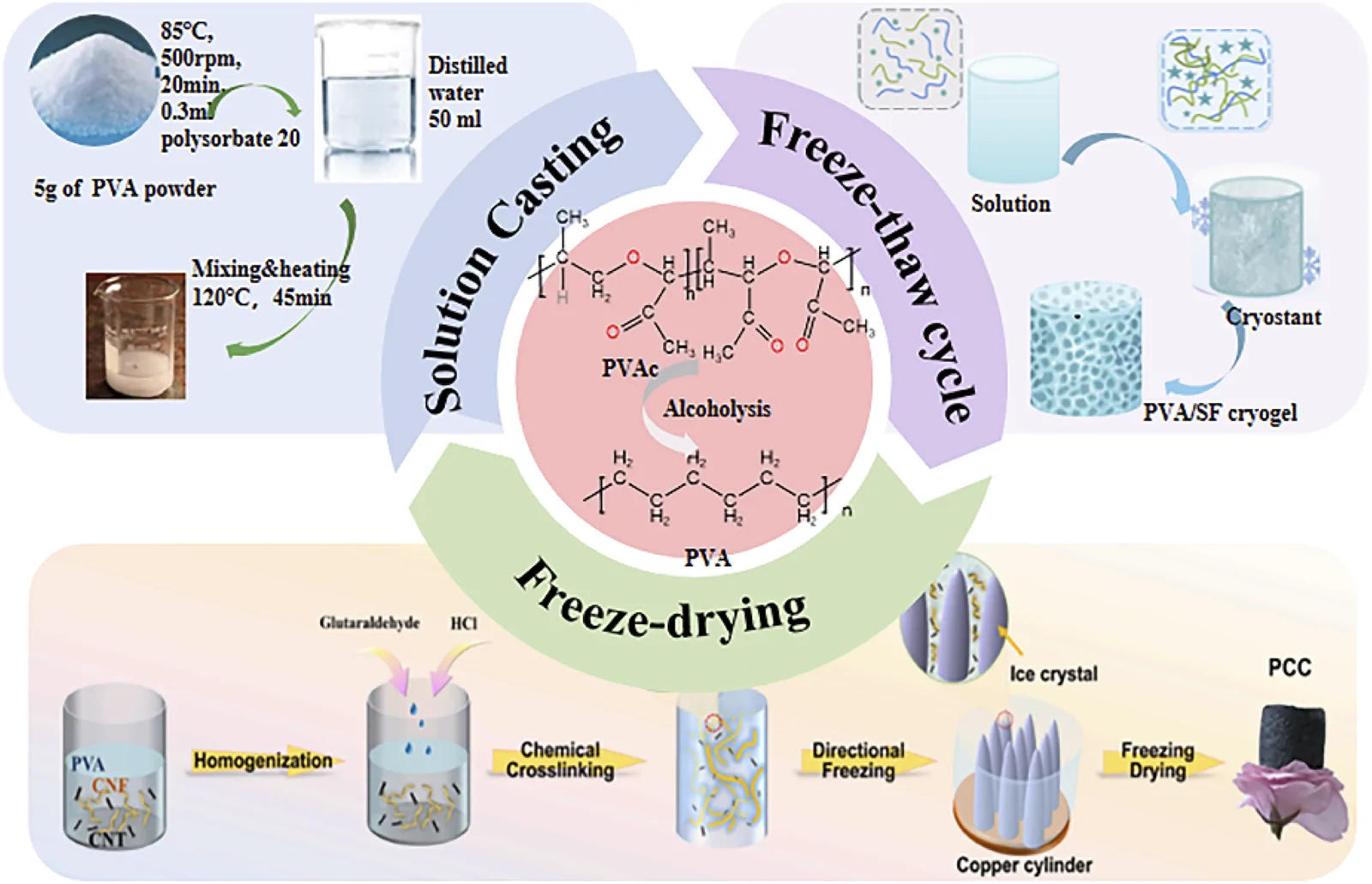
Schematic illustration of the microstructure of PVA-based composites fabricated using three processes—solution casting, freeze-drying and freeze–thaw cycling—and their electromagnetic shielding mechanisms.^45–47^

Solution casting is one of the most commonly used processes for the preparation of PVA films. Tijani *et al.*^45^ systematically investigated the influence of different plasticisers on the properties of PVA/Brazilian palm wax composite films prepared by solution casting, and found that the mechanical properties and water vapour barrier performance of the films could be effectively controlled by selecting different plasticisers such as glycerol, sorbitol and glucose. Similarly, Supriyanto^48^ and colleagues prepared biodegradable PVA composite packaging films containing waste coffee grounds using a water-based solution casting method, demonstrating the environmentally friendly nature of this approach. Hamaamin *et al.*^49^ utilised the solution casting method to prepare PVA films doped with aubergine peel dye, significantly enhancing the material's optical properties. Furthermore, the researchers used a PVA/chitosan blend as the substrate to prepare macroscopic films and microscopic nanofibre materials *via* solution casting and electrospinning techniques, respectively. This study systematically compared the effects of material morphology on physicochemical properties. The results confirmed that, compared with cast films, the nanofibre networks formed by electrospinning not only exposed more active sites (enhancing antioxidant and adsorption properties) but also restricted the movement of polymer chains, thereby exhibiting superior thermal stability and hydrophilicity.^50^ Recent research indicates that real-time synchrotron wide-angle X-ray scattering can reveal the crystallisation kinetics of PVA hydrogel films during the solution casting process, elucidating the influence of the competitive mechanisms between undercooling and supersaturation on the crystallisation process.^51^ In the fabrication of electromagnetic shielding composite films, the solution casting method utilises matrix contraction during solvent evaporation to induce a close, layered arrangement of two-dimensional conductive fillers (such as MXene or graphene), thereby constructing a dense conductive network to enhance electromagnetic wave reflection loss; whereas the microscopic fibre network formed by electrospinning effectively creates internal multi-level heterogeneous interfaces, providing abundant active centres for polarisation loss.

The freeze–thaw cycling method is a classic technique for preparing physically cross-linked PVA hydrogels; it utilises the formation and melting of ice crystals during repeated freeze–thaw cycles to promote physical cross-linking *via* hydrogen bonds between PVA molecular chains. Physical cross-linking avoids the introduction of chemical cross-linking agents, endowing the hydrogel with good biocompatibility. Niu *et al.*^46^ employed the freeze–thaw cycling method to prepare PVA/fibronectin freeze-gels and, by optimising the freeze–thaw parameters, significantly enhanced the mechanical strength of the material, rendering it suitable for cartilage tissue engineering repair. This study demonstrated that, following multiple freeze–thaw cycles, the density of hydrogen-bond cross-links between PVA molecular chains increased significantly, thereby enhancing the mechanical stability of the gel. Furthermore, researchers have combined freeze–thaw physical cross-linking with chemical cross-linking strategies to develop dual-network PVA hydrogels with high strength and high toughness. For example, Lamas *et al.*^52^ prepared PVA/agar dual-network hydrogels using a combination of thermal gelation and freeze–thaw cycles, and achieved excellent mechanical properties by regulating the synergy between the two gelation mechanisms. With regard to the chemical cross-linking modification of PVA, Zakrzewska^53^ and colleagues reviewed green cross-linking strategies for eco-friendly PVA-based nanostructured biomaterials, covering a variety of methods including natural cross-linking agents, radiation cross-linking and enzyme-mediated cross-linking. These green cross-linking strategies provide new avenues for the application of PVA in fields such as biomedicine and flexible electronics. This approach, which reconstructs the PVA matrix network by regulating the cross-linking network (through the synergy of physical hydrogen bonds and chemical covalent bonds), not only endows wearable flexible shielding devices with exceptional tensile resistance and self-healing mechanical properties, but also enables the water-rich internal phase and the microscopic cross-linked network to significantly enhance the absorption and attenuation of electromagnetic wave energy *via* a dipole polarisation mechanism.

Freeze-drying technology is the core method for preparing PVA aerogel materials and is also key to achieving complex microstructural engineering. By precisely controlling the nucleation and growth direction of ice crystals (*e.g. via* unidirectional or bidirectional ice-template methods), researchers are able to use PVA as a flexible scaffold to induce the macroscopic three-dimensional assembly of two-dimensional materials (such as MXene or graphene). This method not only enables the construction of conventional isotropic porous networks but also allows the design of highly ordered biomimetic microstructures (such as a layered brick-and-mortar structure mimicking mother-of-pearl,^54^ or an oriented multi-level pore structure resembling the stomach of a butterfly^55^). Such microstructural engineering greatly extends the propagation and dissipation paths of electromagnetic waves within the material, achieving a dual leap in mechanical resilience and electromagnetic shielding performance at ultra-low densities. Yu *et al.*^56^ employed a dual-template strategy using ice templates, combined with microbubble engineering, to construct superelastic, flame-retardant aerogels with hierarchical porous structures. In this approach, PVA served as the scaffold material, forming a three-dimensional porous network *via* freeze-drying, which exhibited excellent mechanical resilience and an ultra-low thermal conductivity. Yan *et al.*^57^ used PVA and alginate as precursors to prepare composite aerogels with super-wetting properties *via* freeze-drying combined with chemical vapour deposition (CVD) post-treatment, achieving highly efficient oil–water separation and further expanding the scope of application for PVA aerogels. Other researchers constructed topological network structures by encapsulating PVA around a cellulose nanofibre scaffold and loading carbon nanotubes as light-absorbing materials; the resulting aerogels demonstrated excellent performance in the fields of solar-driven water evaporation and adsorption.^47^ The aforementioned studies indicate that freeze-drying technology endows PVA composites with excellent three-dimensional continuous porous scaffolds or three-dimensional topological networks. Once high-conductivity nanofillers are introduced into these anisotropic or multi-level hierarchical porous skeletal structures, they can form high-density three-dimensional microwave reflection/refraction interfaces at ultra-low densities. This causes electromagnetic waves transmitted into the interior of the aerogel to undergo multiple internal reflections between the microscopic pore walls, thereby efficiently converting microwave energy into thermal energy for dissipation.

In summary, the methods for preparing polyvinyl alcohol have evolved from the traditional solution casting method to a multidimensional technical system encompassing physical cross-linking (freeze–thaw cycles), chemical cross-linking, freeze-drying and controlled polymerisation. This has led to the development of strategies for preparing composite materials in various forms, such as films, hydrogels, aerogels and fibres, providing a wealth of technical options for the application of PVA in different fields.

## 3 Theoretical basis of EMI shielding in PVA systems

### 3.1 General principles of EMI shielding

The essence of electromagnetic shielding lies in the interaction between electromagnetic waves and the shielding material, which causes the energy of the electromagnetic waves to attenuate.^40^ The main theories currently used to explain the mechanism of electromagnetic interference shielding include the eddy current theory, the electromagnetic field theory and the transmission line theory.^58,59^ Of these, the transmission line theory is the most widely adopted due to its high accuracy and ease of calculation and understanding. According to Schelkunoff's transmission line theory, when electromagnetic waves in air incident upon the surface of an electromagnetic shielding material, a portion of the waves is reflected at the interface due to impedance mismatch between the material and the air, whilst the remainder is transmitted into the interior of the material. The electromagnetic waves undergo multiple reflections within the material, thereby dissipating the electromagnetic energy. Furthermore, microstructures within the material, such as pores and interfaces, can further enhance the dissipation process. Ultimately, any residual electromagnetic waves that have not been dissipated are transmitted out of the material.^60–62^ The mechanism of electromagnetic shielding is illustrated in Fig. 3.

**Fig. 3:**
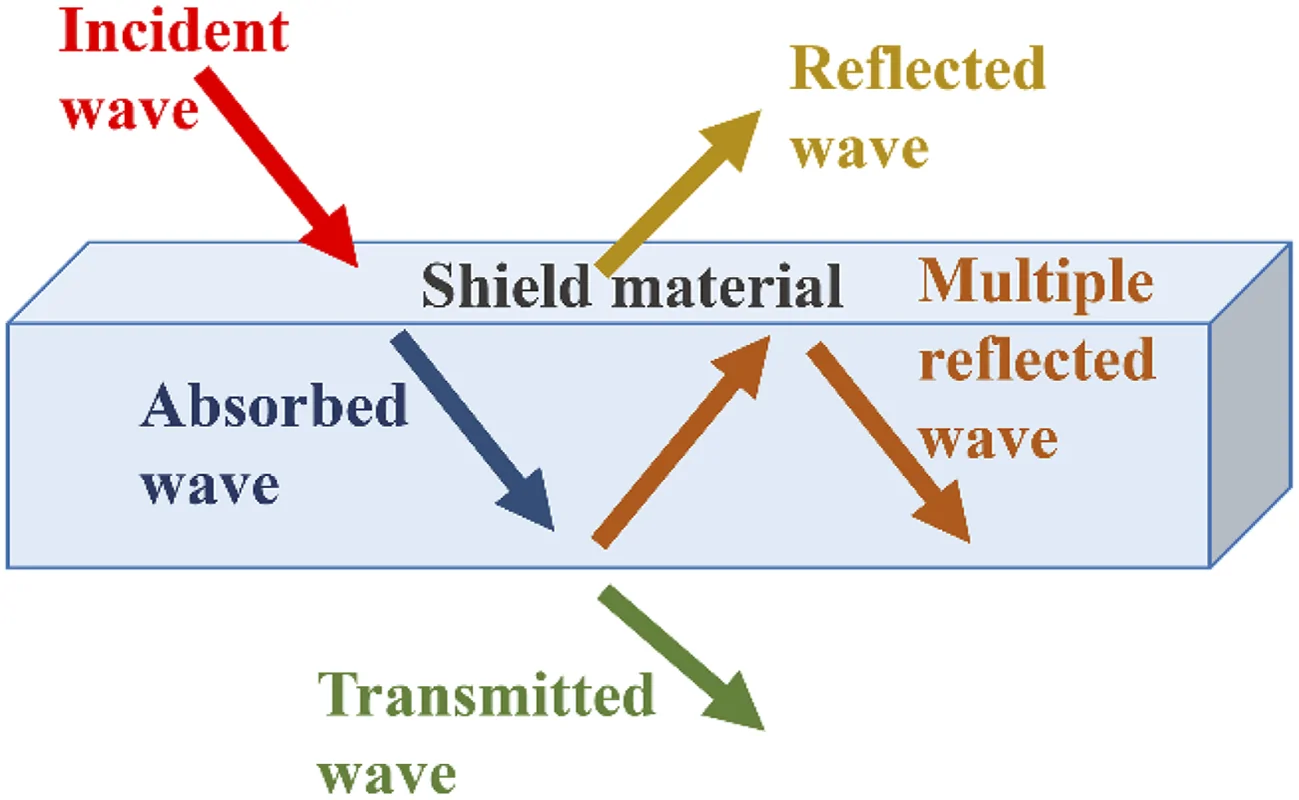
Schematic of EMI shielding mechanisms.

The parameter used to measure a material's electromagnetic shielding performance is Electromagnetic Interference Shielding Effectiveness (EMI SE), expressed in decibels (dB).^61^ It is generally accepted that a higher SE value indicates better electromagnetic shielding performance of the material. EMI SE comprises three components: reflection loss SE_R_, absorption loss SE_A_ and multiple reflection loss SE_M_, as shown in eqn (1-1)–(1-5).^62,63^1-1
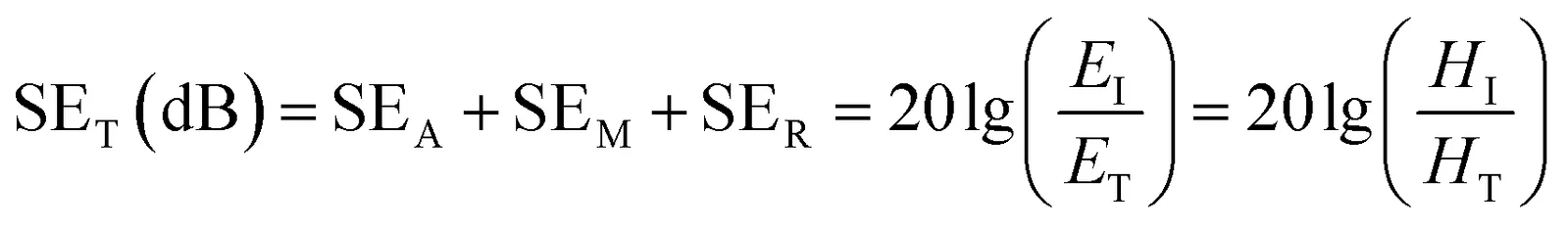
1-2
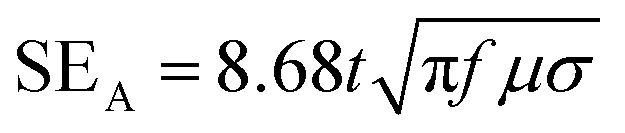
1-3
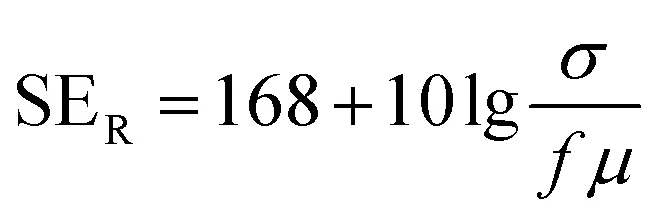
1-4
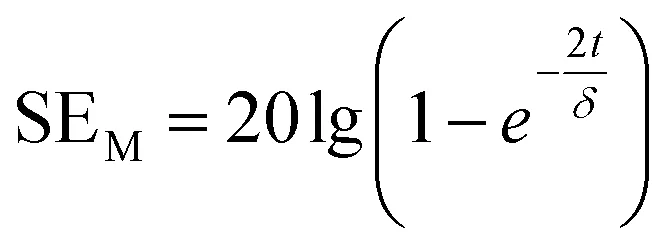
1-5
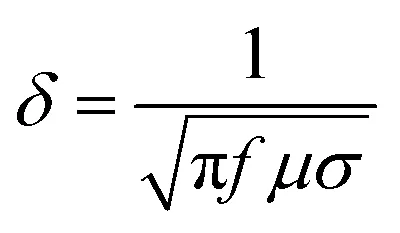
Here, *E*_I_ and *E*_T_ denote the electric field intensities of the incident and transmitted electromagnetic waves, respectively; *H*_I_ and *H*_T_ denote the magnetic field intensities of the incident and transmitted electromagnetic waves, respectively; *f* is the frequency of the incident electromagnetic wave; *µ* and *σ* represent the magnetic permeability and electrical conductivity of the material, respectively; *t* is the thickness of the sample; and *δ* is the skin depth. In eqn (1-2), the constant 8.68 arises from the conversion of the exponential attenuation factor to decibels, *i.e.*, 20/ln(10) ≈ 8.686. In eqn (1-3), the constant 168 originates from the free-space intrinsic impedance, *i.e.*, 20 lg(*η*_0_/2π) ≈ 168, where *η*_0_ ≈ 377 Ω. In PVA-based porous materials (such as aerogels or foams), it is worth noting that, according to classical transmission line theory, the multiple reflection loss component SE_M_ is mathematically expressed as tending towards zero. When the material thickness *t* is much greater than its skin depth *δ*, the electromagnetic waves entering the interior of the material are completely dissipated before reaching the rear surface; in this case, SE_M_ can be neglected. Only in ultra-thin or ultra-low-conductivity systems does SE_M_ manifest due to the disordered bouncing and leakage of electromagnetic waves between the inner and outer surfaces; essentially, this degrades the overall shielding performance. The abundant pores within PVA materials, the three-dimensional continuous framework, and the heterogeneous interfaces formed between PVA and fillers not only enhance interfacial polarisation loss but also cause electromagnetic waves transmitted into the material to undergo countless reflections and scatter within microscopic channels, ultimately being dissipated and converted into thermal energy.^63,64^ When the material's SE_T_ exceeds 20 dB, it is capable of meeting the basic requirements for commercial applications.^65^

Typically, the EMI SE of a material is determined by measuring *S*-parameters using a vector network analyser (VNA). *S*-parameters are typically measured using the waveguide method, the coaxial method or the anechoic chamber method.^62^ Based on the two-port parameters *S*_11_, *S*_12_, *S*_21_ and *S*_22_, the reflection coefficient (*R*), absorption coefficient (*A*), transmission coefficient (*T*), SE_A_, SE_R_ and SE_T_ are calculated respectively, as shown in eqn (1-6)–(1-10):^18,62^

1-6 *R* = |*S*_11_|^2^ = |*S*_22_|^2^

1-7 *T* = |*S*_12_|^2^ = |*S*_21_|^2^

1-8 *A* = 1 − *R* − *T*

1-9
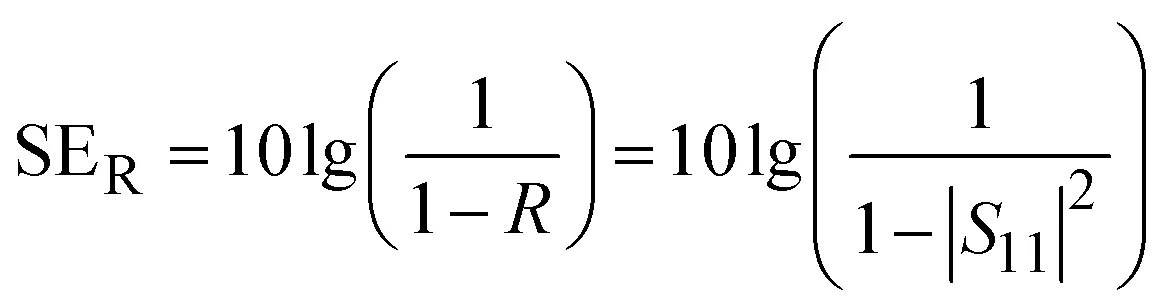
1-10
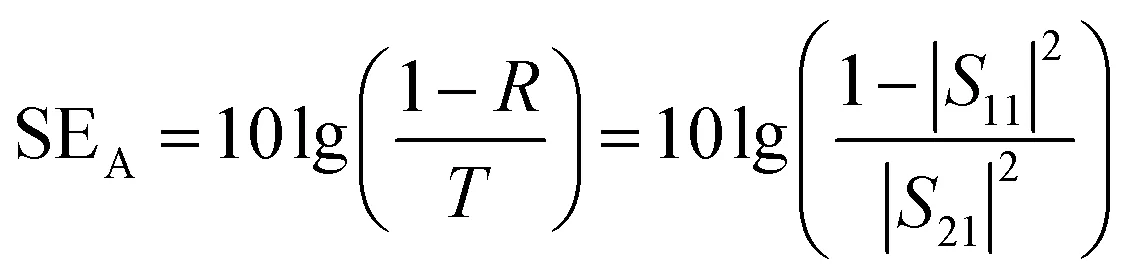
Here, *R* is the power reflection coefficient, *T* is the power transmission coefficient, and *A* is the power absorption coefficient, defined as the fraction of incident electromagnetic wave power dissipated within the material. *S*_11_ and *S*_22_ are the reflection coefficients at ports 1 and 2, respectively (*S*_11_ = *S*_22_ for a symmetric two-port system), while *S*_12_ and *S*_21_ are the transmission coefficients from port 2 to port 1 and from port 1 to port 2, respectively (*S*_12_ = *S*_21_ for a reciprocal system). The electromagnetic shielding mechanism of a material is determined by analysing its reflection coefficient and absorption coefficient. By comparing the values of *R* and *A*: when *R* > 0.5 and *R* > *A*, the material's shielding mechanism is predominantly based on reflection; conversely, when *A* > 0.5 and *A* > *R*, the material's shielding mechanism is predominantly based on absorption.^66^

### 3.2 Impedance matching in PVA matrices

Impedance mismatch is the fundamental cause of strong surface reflection in highly conductive composites. In PVA-based systems, this issue can be mitigated by the ‘dielectric dilution’ effect inherent to the matrix itself; however, the specific mechanism by which this occurs is not uniform but depends on the macroscopic morphology of the material. The physical mechanism underlying the ‘dielectric dilution’ effect in PVA-based composites varies according to the material's macroscopic morphology and can be broadly categorised into two types: solid-state systems and hydrogel systems.

For solid-state systems (films, aerogels, foams), impedance matching relies primarily on PVA's inherently low dielectric constant.^67^ At the microscopic scale, the crystalline or amorphous regions of PVA act as insulating layers, separating the highly conductive fillers (such as MXene and CNTs) from one another.^68^ This spatial isolation effect is equivalent to introducing a set of low-dielectric layers between the conductive networks, which reduces the equivalent dielectric constant of the composite as a whole, thereby mitigating the impedance mismatch caused by high conductivity.^67^

The logic behind impedance matching in hydrogel systems is different, with the key lying in the regulation of the dielectric response of the water molecules within them.^69,70^ From a microwave dielectric perspective, the abundant free water present in hydrogels is the primary source of strong interface reflection.^71^ The three-dimensional hydrophilic network of PVA can, through hydrogen bonding, convert a portion of the free water into intermediate water or bound water; the dielectric constants and relaxation frequencies of these two types of water are significantly lower than those of free water, thereby helping to reduce the material's overall apparent dielectric constant and gradually optimise the input impedance.^72,73^

It must be noted that these two pathways are not entirely distinct. Taking the PVA-MXene hydrogel as an example, the MXene flakes act both as centres of conductive loss and as physical cross-linking points that enhance the confinement of water molecule motion, effectively mobilising both dilution mechanisms simultaneously.^24^ The ultimate effect of both pathways is to bring the material's input impedance *Z*_in_ closer to the free-space impedance *Z*_0_; however, there is a trade-off between them: a one-sided pursuit of crystallinity dilution sacrifices the continuity of the conductive network, while excessive bound water reduces the efficiency of dielectric loss.^73,74^ Consequently, the crux of the matter lies in finding the optimal balance between the proportion of crystalline regions or the content of bound water and the continuity of the conductive network; this is also the core physical challenge in realising ‘low-reflection, high-absorption’ green shielding materials.

## 4 Recent advances in polyvinyl alcohol-based electromagnetic shielding composites

In recent years, with the rapid evolution of 5G communication technology and a wide range of portable electronic devices, we are living in an increasingly complex electromagnetic environment. Among numerous polymer matrices, polyvinyl alcohol (PVA) has demonstrated significant advantages in the development of functional composites due to its excellent biocompatibility and the abundance of hydroxyl functional groups in its molecular chains.^75^ These functional groups can effectively bridge high-conductivity fillers such as MXene, forming hydrogen bonds and other interactions that reduce the agglomeration of functional fillers.^76^ Through simple physical blending or chemical modification, PVA can be combined with a variety of conductive fillers to construct composite material systems that possess both good mechanical properties and excellent electromagnetic shielding performance.^77,78^ In terms of the forms of materials used, research into the application of PVA in the field of electromagnetic shielding has primarily focused on films, aerogels, foams and hydrogels^79–83^ (as shown in Fig. 4).

**Fig. 4:**
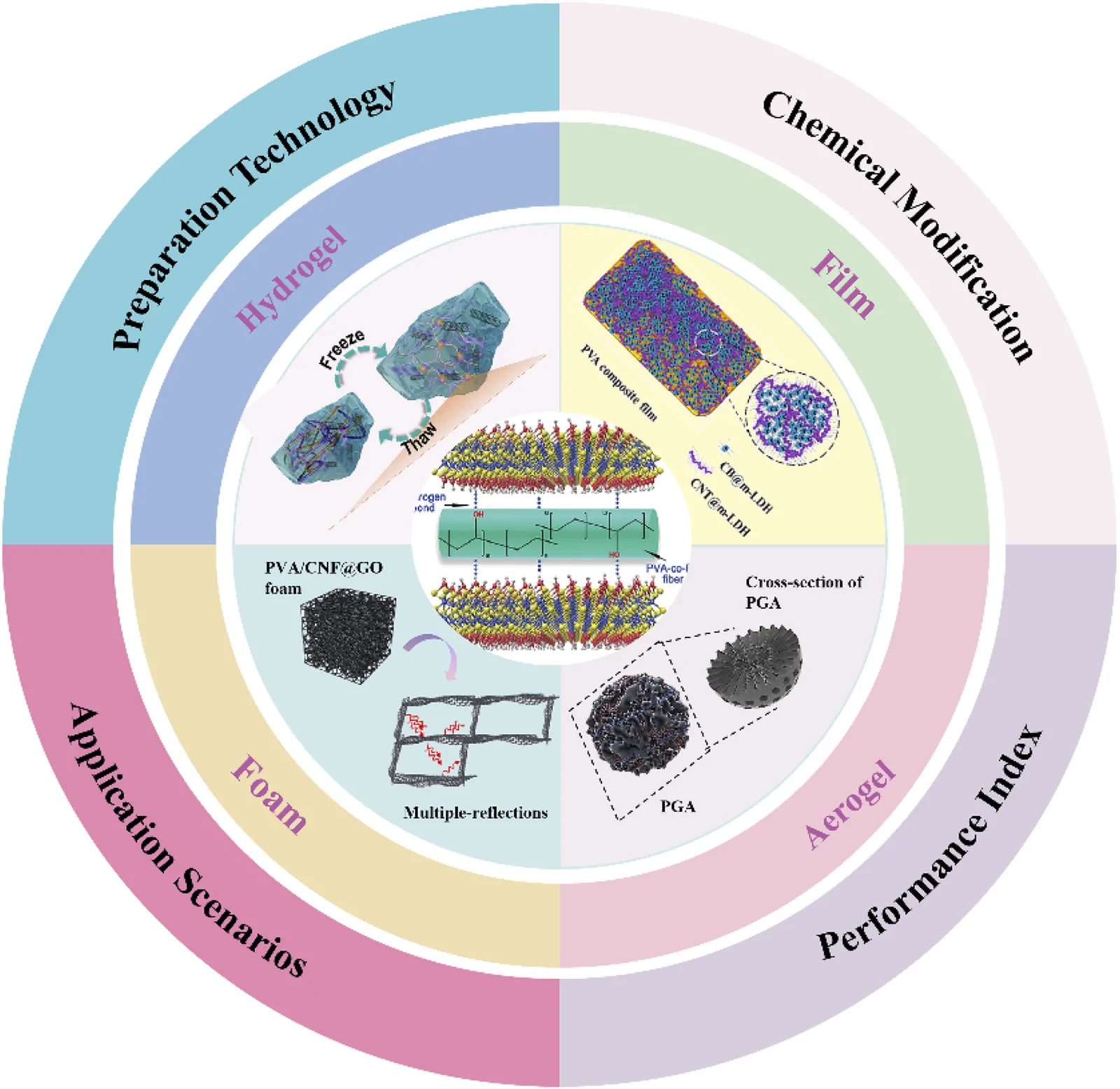
Research areas for polyvinyl alcohol (PVA)-based electromagnetic shielding materials.^79–83^

### 4.1 Current status of research on polyvinyl alcohol-based electromagnetic shielding films

In the development of flexible, ultra-thin electromagnetic shielding films, PVA has emerged as an ideal flexible polymer matrix due to its excellent film-forming properties and exceptional transparency.^84,85^ Its water-soluble nature and abundance of hydroxyl groups^86^ not only endow the matrix with excellent dispersion and forming capabilities for two-dimensional conductive nanomaterials (such as MXene and graphene), but also lay a solid chemical foundation for stress transfer at heterointerfaces and the formation of polarisation networks.^87,88^

The solution casting method has become the mainstream processing technique for PVA shielding films due to its simplicity of operation and suitability for large-scale production.^88,89^ Shen *et al.*^90^ prepared a ternary MXene/graphene@ferric oxide/PVA composite film with high flexibility and good electromagnetic shielding performance *via* a simple solution casting process. The conductive fillers, MXene and graphene, promote the dielectric loss of electromagnetic waves, whilst the magnetic ferric oxide particles facilitate magnetic loss; together, these three components achieve effective attenuation of electromagnetic waves. At a thickness of 1 mm, this film achieves an electromagnetic shielding efficiency of 36 dB in the X-band, with an elongation at break exceeding 160%, demonstrating excellent combined ‘electromagnetic–mechanical’ performance.

In addition to multi-component hybridisation strategies, it is equally crucial to investigate the penetration threshold and mechanical trade-off effects of single high-conductivity fillers in the matrix. Muhammad Irfan Jahanger *et al.*^43^ employed a solution-casting method to prepare ultra-thin, high-strength, and highly flexible MXene-PVA composite films with different MXene contents (20, 40, 60 and 80 wt%) (as shown in Fig. 5). At an MXene content of 60 wt%, the composite film exhibited optimal mechanical properties: a tensile strength of 56.0 MPa, a strain at break of 2.2%, and a Young's modulus of 6.7 GPa. Meanwhile, when the MXene content reached 80 wt%, the electromagnetic interference (EMI) shielding performance peaked, with a shielding efficiency of 34.80 dB.

**Fig. 5:**
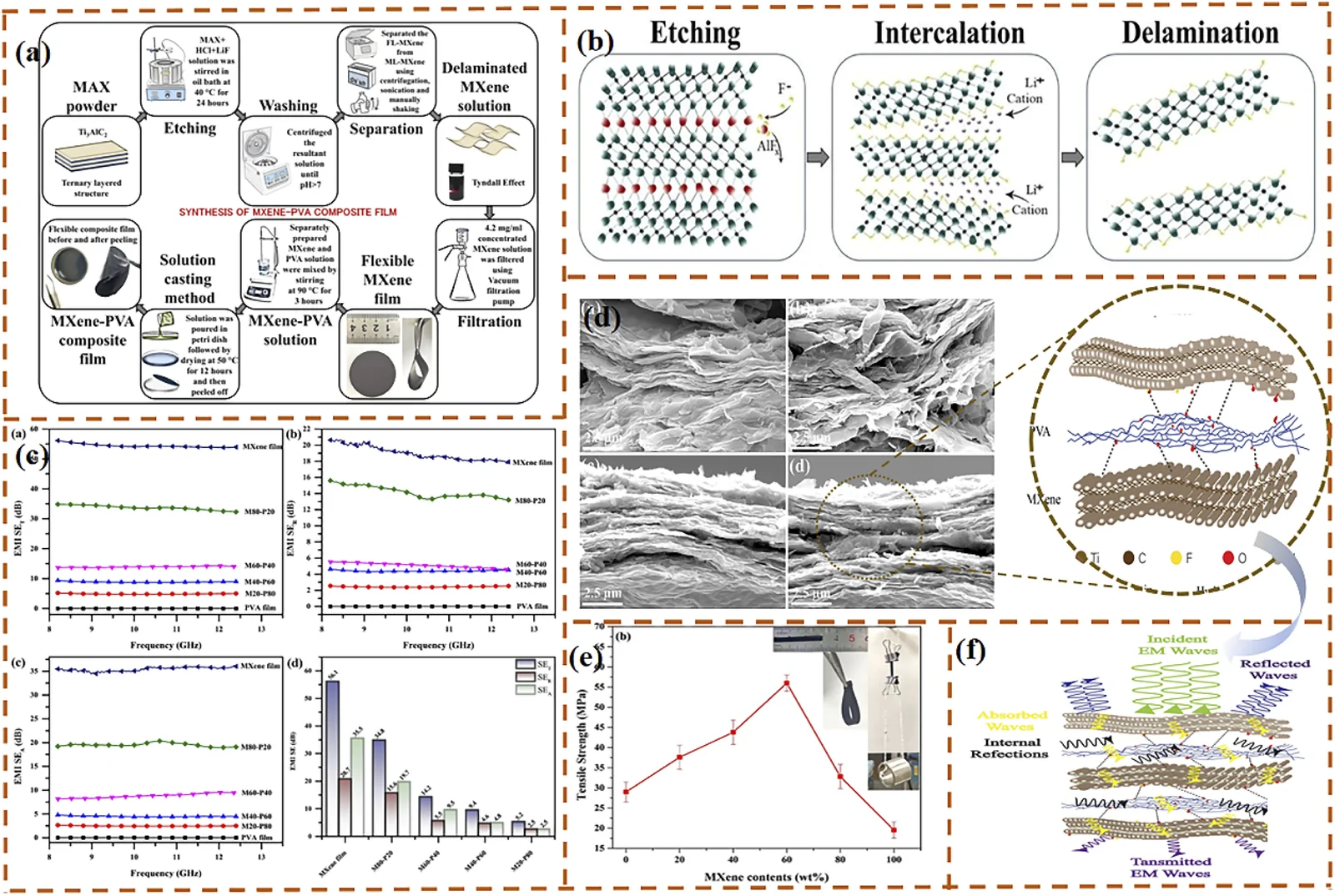
Electromagnetic interference (EMI) shielding performance and shielding mechanism analysis of the self-supporting MXene–PVA composite films presented in panels (a)–(f). Reproduced from ref. 43 with permission from Elsevier, Copyright 2025.

In practical working environments, the miniaturisation and increasing power density of electronic devices have led to demands for materials with multifunctional properties, such as high thermal conductivity and intrinsic flame retardancy. Driven by these requirements, the macroscopic design of spatial structures (such as multilayer alternating structures and asymmetric gradient structures) has emerged as an effective approach to overcoming the performance bottlenecks of homogeneous blending systems. Jin *et al.*^87^ prepared alternating multilayer PVA/MXene films with both high electromagnetic shielding and thermal conductivity using a multilayer casting method. The continuous MXene layers provide a dense network for thermal and electrical conductivity, enabling the multilayer films to exhibit excellent electromagnetic shielding and thermal conductivity simultaneously. A 27 µm-thick PVA/MXene multilayer film (containing a total of 19.5 wt% MXene) exhibited a conductivity of 716 S m^−1^, an EMI SE value of 44.4 dB, and a high thermal conductivity of 4.57 W mK^−1^—an improvement of nearly 23-fold compared to pure PVA. Furthermore, the researchers investigated the flame-retardant properties of the films and found that the thicker the MXene layer or the higher the MXene content, the better the film structure was maintained. This indicates that MXene content plays a key role in the flame-retardant properties of PVA/MXene multilayer films, thereby conferring excellent flame-retardant characteristics to the material.

PVA, acting as an insulating matrix, effectively isolates the highly conductive MXene flakes at the microscopic scale. At the surface of the composite, the enrichment of PVA significantly reduces the macroscopic surface DC conductivity, mitigating the impedance discontinuity at the air–material interface and enabling electromagnetic waves to penetrate smoothly into the interior of the material rather than being directly reflected at the surface. Within the material, PVA forms hydrogen bonds with the surface functional groups of MXene *via* its abundant hydroxyl groups, thereby suppressing van der Waals-induced agglomeration between MXene layers and facilitating the formation of a uniform three-dimensional distribution. Although PVA is an insulating phase, the thickness of the insulating layer it forms between adjacent MXene layers is typically on the nanometre scale (1–10 nm). At this scale, electrons in an alternating electromagnetic field can overcome this insulating barrier *via* the quantum tunnelling effect; simultaneously, the ‘MXene-PVA-MXene’ structure can be regarded as an array of microcapacitors in an alternating electric field, generating significant interfacial polarisation and dielectric relaxation losses. Consequently, even when the direct-current conductive path is partially interrupted, the material is still able to maintain efficient attenuation of electromagnetic waves through the two aforementioned alternating-current loss mechanisms. In addition to PVA-based films, strategies for constructing conductive networks in other polymer matrices are also worth learning from.

### 4.2 Current status of research on polyvinyl alcohol-based electromagnetic shielding aerogels and foams

Aerogels and foam materials with three-dimensional porous structures can meet the demand for lightweight, highly efficient electromagnetic shielding materials in fields such as aerospace.^91,92^ The hydroxyl groups on PVA molecular chains can form hydrogen bonds with conductive fillers, enabling the preparation of low-density, high-porosity conductive scaffolds *via* freeze-drying, the template method and foaming techniques.^20,93^ The porous structure can extend the propagation path of electromagnetic waves within the material,^94,95^ three-dimensional, highly developed porous frameworks possess a vast number of heterogeneous interfaces; when incident microwaves penetrate into the interior of the pores, the pore walls repeatedly induce refraction, scattering and microscopic multiple reflections of the microwaves. The microscopic multiple reflections induced by the porous structure effectively extend the propagation path of electromagnetic waves, ultimately multiplying in the form of bulk absorption loss, thereby achieving ‘high absorption, low reflection’ green shielding characteristics at ultra-low densities.^96^

Yan *et al.*^97^ prepared a PEG/Ni-F/PPy@CNF-PVA (PPCN) phase-change composite foam with energy storage and electromagnetic shielding properties using freeze-drying and vacuum-assisted adsorption processes. PPCN exhibited good stability and high energy storage density, with a melting enthalpy as high as 126.18 J g^−1^ and a relative enthalpy efficiency exceeding 99%, whilst demonstrating excellent electromagnetic shielding performance in the X-band (EMI SE value as high as 110.37 dB). In contrast to the former preparation process, Song *et al.*^98^ used a foaming method to prepare a lightweight, recyclable, porous PVA fibre/silver-coated carbon fibre (CF@Ag) electromagnetic shielding composite foam, which exhibited an electromagnetic shielding efficiency of 86.1 dB and a specific shielding efficiency of 5100 dB cm^3^ g^−1^. As shown in Fig. 6, this foam retains effective shielding performance even after multiple compression cycles and thermal treatment, which precisely meets the requirements for materials used in the aerospace sector.

**Fig. 6:**
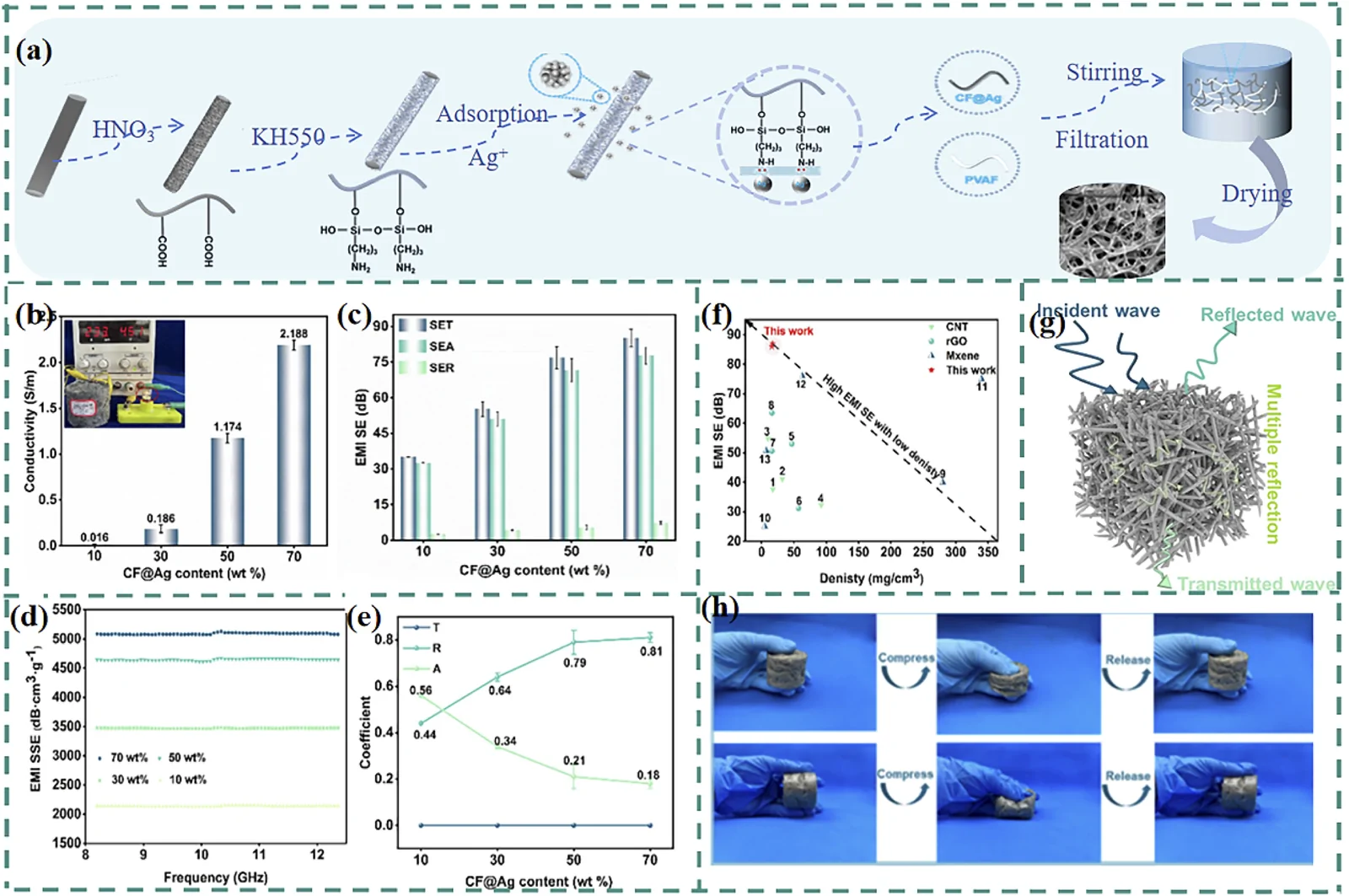
Preparation process, morphology, electromagnetic shielding performance, and mechanical properties characterization of recyclable PVA fibre/silver-coated carbon fibre (CF@Ag) composite foam materials presented in panels (a)–(h). Adapted from ref. 98 with permission from Elsevier, Copyright 2025.

Chen *et al.*^41^ prepared vertically aligned, doubly cross-linked MXene/PVA composite aerogel *via* an ice-template unidirectional freezing process followed by glutaraldehyde (GA) post-treatment. In the X-band, with an MXene loading of just 3.81 vol%, this aerogel achieved an electromagnetic shielding efficiency of 32.6 dB. Furthermore, GA not only enhances the structural stability of the composite aerogel but also confers excellent flame-retardant properties. The effective utilisation of waste materials in electromagnetic interference (EMI) shielding has been widely reported. Zhang *et al.*^99^ prepared a novel integrated leather solid waste (LSW)/PVA/polyaniline (PANI) aerogel that combines high mechanical strength, flame retardancy and electromagnetic shielding performance. The material's EMI SE value exceeded 40 dB, with a specific shielding efficiency of 73.0 dB cm^3^ g^−1^. As LSW contains a large amount of nitrogen, a nitrogen-based flame-retardant mechanism was established, raising the aerogel's limiting oxygen index to 32.0% at a thickness of 2.5 mm; this also provides new insights into the development of biomass aerogels that combine high performance, environmental friendliness and economic viability.

### 4.3 Current status of research on polyvinyl alcohol-based electromagnetic shielding hydrogels

Hydrogels consist of three-dimensional hydrophilic polymer networks that encapsulate a large volume of aqueous phase within a porous structure, endowing them with exceptional flexibility, elasticity and stretchability.^100,101^ Furthermore, the complex porous network enables multiple reflections of electromagnetic waves, whilst the dipole water molecules within the matrix effectively dissipate incident electromagnetic energy through a polarisation loss mechanism.^102,103^ Owing to their hydrophilic network structure and excellent mechanical ductility, polyvinyl alcohol hydrogels have become ideal flexible matrix materials for electromagnetic shielding.

However, it is worth noting that the high dielectric loss resulting from the high water content in hydrogel systems stems largely from the dipole polarisation response of intrinsic water molecules in the microwave frequency range, which often leads researchers to overlook the intrinsic contribution of the polymer matrix itself.

In fact, the three-dimensional hydrophilic network of PVA exerts a unique ‘spatial tailoring’ effect on the dissipation behaviour of water molecules. According to polymer swelling kinetics, three states of water exist within the hydrogel: unbound free water, weakly bound intermediate water, and strongly bound water that forms strong hydrogen bonds with the hydroxyl groups of PVA segments.^104^ Under an alternating electromagnetic field, free water exhibits an extremely high imaginary part of the complex permittivity (*ε*″), resulting in severe relaxation loss.^105^ Meanwhile, the PVA molecular chains impose spatial steric hindrance on the motion of water molecules *via* the hydrogen-bond network, converting part of the free water into bound water, which leads to a shift in the dipole moment's orientation relaxation time. This regulation of the aqueous phase by the matrix not only optimises impedance matching within the material—thereby preventing severe reflection of electromagnetic waves at the material's surface caused by the excessively high dielectric constant of pure water—but also utilises the synergistic interfacial polarisation of confined water molecules to create highly efficient microwave attenuation centres within the matrix.^106^ Consequently, the core of the shielding design for PVA hydrogels lies not only in the construction of the filler network, but also in utilising the steric hindrance effect of bound water to effectively regulate the excessively high dielectric constant, thereby optimising impedance matching, whilst retaining an appropriate amount of free water for high-frequency dipole dissipation,^107,108^ in order to achieve perfect synergy between impedance matching and intrinsic dielectric loss.^26,109^

Wang *et al.*^100^ prepared PVA composite hydrogels reinforced with MXene and aramid nanofibres (ANFs) *via* solution exchange, freeze–thaw cycles and salt precipitation. These composite hydrogels achieved an X-band electromagnetic interference (EMI) shielding efficiency of 64.5 dB at an extremely low filler concentration (1.5 wt% MXene by mass). Interestingly, this composite hydrogel is also capable of encoding and encrypting information based on different signals, opening up broad prospects for the fields of smart sensing and wearable devices. Silver nanowires are widely used in the field of electromagnetic shielding due to their excellent conductivity,^110,111^ however, they are prone to agglomeration within the matrix, which affects their performance. As shown in Fig. 7, Zhu *et al.*^42^ prepared hydroxyl-terminated silver nanowires (M-AgNWs) by modifying silver nanowires with bis(2-hydroxyethyl) disulphide, and then incorporated them into a PVA/borax hydrogel system to produce a PB/M-AgNWs hydrogel with self-healing properties and excellent electromagnetic shielding performance. In this study, an 8 mm-thick PB/M-AgNWs (0.1%) hydrogel exhibited an EMI SE of 65.10 dB, with an attenuation rate of 99.9999% for incident electromagnetic waves. Xu *et al.*^112^ prepared MXene/PVA/polyacrylic acid (PAA) composite hydrogels using a freeze–thaw cycling method; when the mass fraction of MXene was 10 wt%, a 2 mm-thick MXene/PVA/PAA hydrogel achieved an electromagnetic shielding efficiency of 33.6 dB.

**Fig. 7:**
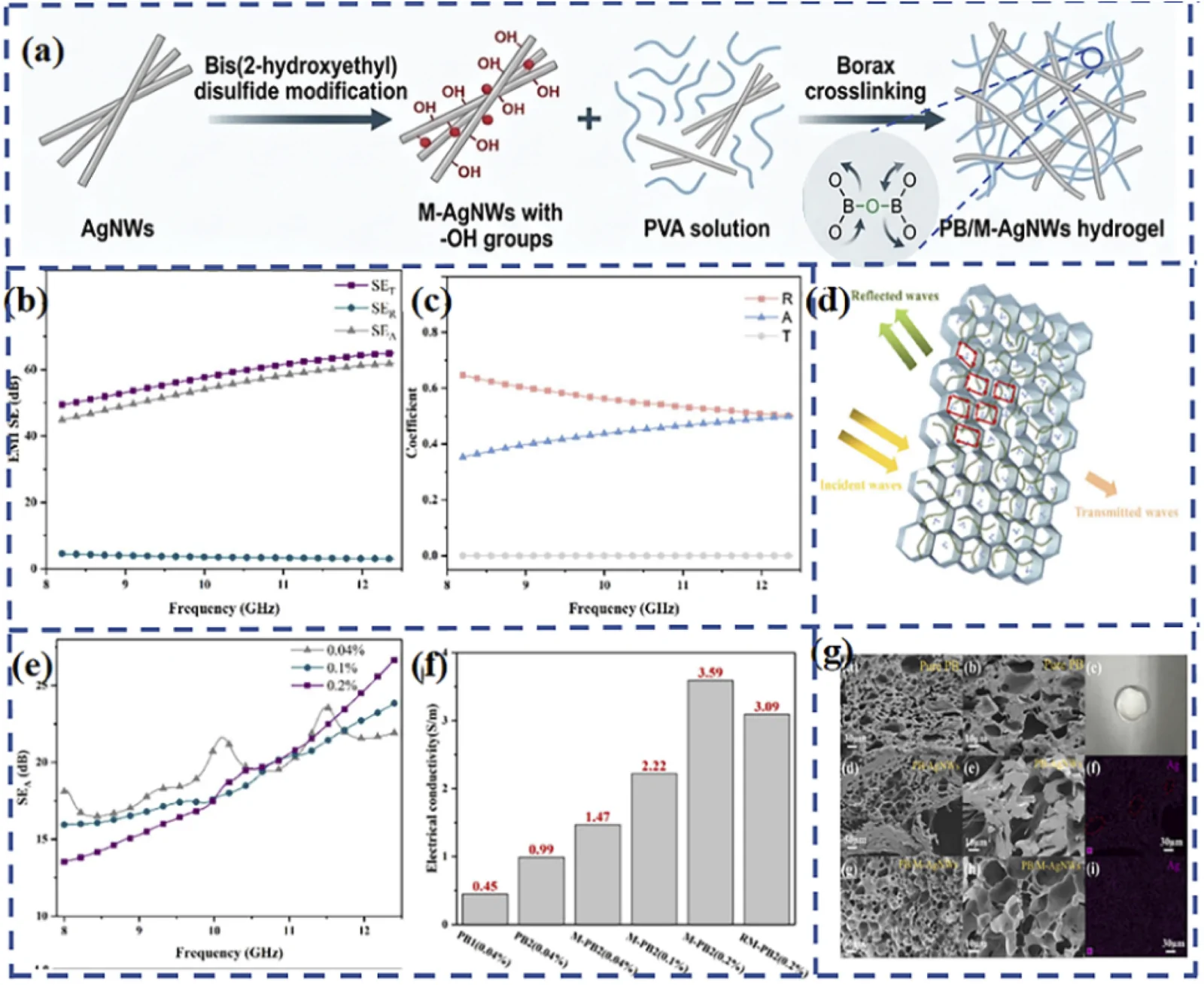
Schematic illustration of the preparation mechanism, microstructure, and electromagnetic shielding performance characterization of the PVA/borax/modified silver nanowires (PB/M-AgNWs) composite hydrogel, shown in panels (a)–(g). This hydrogel exhibits self-healing properties and high electrical conductivity. Reproduced from ref. 42 with permission from Elsevier, Copyright 2025.

Numerous studies have demonstrated that hydrogels possess good hydrophilicity and flexibility, whilst Lu *et al.*^101^ prepared a polypyrrole nanotube-polyethylene glycol-polyvinyl alcohol (PPPg) hydrophobic hydrogel with excellent electromagnetic shielding performance, high flexibility, environmental friendliness and corrosion resistance *via* hydrogen plasma treatment. At a thickness of 2 mm, this hydrogel achieved an EMI SE of 21 dB in the X-band, meeting commercial application requirements. Furthermore, the PPPg exhibited an elastic deformation of 100.9% under a pressure of 2.28 MPa, demonstrating excellent flexibility; this holds great promise as a multifunctional flexible nanocomposite for wearable devices requiring high mechanical performance. Based on physical and chemical cross-linking reactions, Li *et al.*^113^ synthesised a multifunctional interpenetrating network PASCM hydrogel composed of PVA, acrylamide, sodium alginate, choline chloride and MXene. In the X-band, the PASCM hydrogel exhibits an EMI SE value of 33 dB and retains approximately 84.67% of its initial stress after 100 loading–unloading cycles under a strain of 600%; its sensitivity factor (GF) reaches 5.8. Such materials hold broad application prospects in fields such as health monitoring, human–machine interaction and electromagnetic protection.

The aforementioned studies indicate that the superior electromagnetic shielding performance of PVA hydrogels is not achieved merely through the simple introduction of conductive fillers; rather, it is primarily due to the synergistic regulation of the dielectric environment and mechanical properties by their unique dual-network structure. The physically cross-linked network formed by freeze–thaw cycles contains a large number of microcrystalline regions and hydrogen bonds.^71^ These dense hydrogen-bonding sites can effectively anchor water molecules, converting them into polarisation-restricted bound water, thereby optimising impedance matching.^114^ Under tensile stress, these hydrogen bonds act as sacrificial bonds and break preferentially to dissipate energy, endowing the material with high tensile strength.^115^ Conversely, chemical covalent bonds provide a rigid, irreversible and robust framework that limits the unlimited swelling of the hydrogel and prevents the breakdown of the three-dimensional continuous conductive network caused by excessive water absorption by conductive fillers (such as MXene).^116^ The physical network modulates the state of water molecules to optimise the dielectric environment, whilst the chemical network stabilises the conductive microstructure to ensure macroscopic conductive loss. During tensile loading, the physical network absorbs mechanical energy through the breaking and re-formation of hydrogen bonds, whilst the chemical network ensures that fillers such as MXene are consistently maintained above the percolation threshold, thereby achieving synchronous stability of the electromagnetic loss mechanism under high tensile conditions.^117,118^ This synergistic design concept of a physical-chemical dual network provides important theoretical guidance for the development of hydrogel materials that combine high mechanical toughness with high shielding performance.

To provide a more intuitive comparison of the performance characteristics of polyvinyl alcohol (PVA)-based composites with different morphologies, Table 1 (ref. 41–43, 87, 90, 97–101, 112 and 113) systematically summarises the preparation processes, filler loading, thickness/density, and corresponding electromagnetic shielding efficiency (EMI SE) and multifunctional applications of PVA-based films, aerogels/foams and hydrogels in recent years.

**Table 1:** Comparison of structural design, filler networks and electromagnetic shielding performance in different PVA-based composite matrices^41–43,87,90,97–101,112,113^

Material form	Conductive filler/loading	Preparation method	Tickness/density	EMI SE (dB)	SSE *t*/SSE	SEA/SET (%)	Key multifunctionalities
Film	Ag@PDA@CNT/PVA	Solution casting	0.5 mm	42.75	1193.8 dB cm^2^ g^−1^	75.7	High flexibility
Film	MXene80 wt%/PVA20 wt%	Solution casting	7.0 µm	34.8	24 051.42 dB cm^2^ g^−1^	56.60	High strength & free-standing
Film	MXene/19.5 wt%	Multilayer casting	27 µm	44.4	9343 dB cm^2^ g^−1^	81.30	Thermal conductivity/flame retardancy
Foam	PEG/Ni-F/PPy@CNF	Freeze-drying & vacuum adsorption	2.0 mm	110.37	—	78.50	Energy storage, thermal stability
Foam	PVA/CF70 wt%@Ag	Foaming method	16.7 mg cm^−3^	86.1	5100 dB cm^3^ g^−1^	91.60	Recyclability, excellent mechanical cyclic stability
Aerogel	MXene 2.75 vol%/PVA	Ice-template unidirectional freeze-drying & GA crosslinking	0.1221 g cm^−3^	32.3	269 dB cm^3^ g^−1^	89.20	Flame retardancy, thermal camouflage
Aerogel	LSW/PANI	Freeze-drying	2.5 mm	42.00	73 dB cm^3^ g^−1^	97.00	Mechanical robustness, eco-friendly, flame retardancy
Hydrogel	PVA/MXene1.5 wt%/ANFs	Solution exchange & freeze–thaw & salting-out	3.0 mm	52.00	—	86.00	Information coding/encryption, motion sensing
Hydrogel	Modified AgNWs (M-AgNWs)/0.1 wt%	Borax crosslinking	8.0 mm	65.1	—	≈86	Self-healing, high conductivity
Hydrogel	MXene10 wt%/PAA	Freeze–thaw cycles	2.0 mm	33.6	—	79.20	High flexibility & stretchability
Hydrogel	Polypyrrole nanotubes/8 wt%	Hydrogen plasma treatment	2.0 mm	21.00	—	—	Hydrophobicity, anti-corrosion
Hydrogel	MXene/PAM/AC	Physical–chemical crosslinking	1.5 mm	33.00	—	84.50	Interpenetrating network, health monitoring

As shown in Table 1, the control of microstructure and the design of spatial structure have a decisive influence on the shielding performance and multifunctionality of PVA-based composites. In the case of dense film materials,^77,81,82^ whilst their macroscopic mechanical strength and electrical conductivity are generally high, they often face issues such as high penetration thresholds and high filler loading (up to 80 wt%). Consequently, complex alternating multilayer or asymmetric structural designs are typically required to balance the trade-off between mechanical flexibility and conductive loss. Conversely, by introducing three-dimensional porous structures, the twisted and interconnected pore walls within the material cause incident electromagnetic waves to undergo severe multiple reflection losses. This mechanism enables porous scaffolds to achieve extremely high electromagnetic shielding performance^88–90^ with extremely low filler loading and ultra-low density.

In light of this, the unique spatial engineering significance of the PVA matrix lies in its ability to utilise its abundant side-chain hydroxyl groups to perform ‘dielectric dilution’ and ‘spatial tailoring’ of the conductive network. By finely tuning the physical/chemical cross-linking density,^119^ converting a portion of the free water—which exhibits high dielectric response—into confined water not only reduces the material's surface dielectric constant and opens up impedance-matching pathways,^120^ but also ensures that microwaves penetrate smoothly into the deeper layers of the material. Consequently, the residual free water's dipole relaxation,^24^ interfacial polarisation and porous scattering are utilised to efficiently convert microwave energy into thermal dissipation.^121^ This balanced design,^122,123^ which combines ‘low reflection’ with ‘strong absorption’, is the core concept behind high-performance, environmentally friendly PVA electromagnetic shielding materials.

## 5 Summary and outlook

This paper provides a systematic review of the latest research progress on polyvinyl alcohol (PVA)-based electromagnetic shielding materials, focusing on the structure–property relationships and shielding mechanisms in three typical macroscopic forms: films, aerogels/foams and hydrogels. Research indicates that the abundant hydroxyl groups on the side chains of PVA—a characteristic of the material—can form strong hydrogen-bond networks with highly reactive one-dimensional and two-dimensional conductive nanofillers, such as MXene and carbon nanotubes, thereby enabling the efficient and uniform dispersion of inorganic fillers within macro- and micro-scale structures and the formation of heterogeneous interfaces; by modulating the three major macroscopic morphologies—films, aerogels/foams and hydrogels—and utilising spatial network engineering (such as alternating multilayers or biomimetic porous structures) to induce ‘multiple internal reflection losses’ in incident electromagnetic waves, the study successfully facilitated a shift in the shielding mechanism from the traditional ‘high-reflection’ type to an environmentally friendly ‘high-absorption’ type; the underlying mechanism by which PVA regulates the internal water state (free water and bound water) through the density of physical and chemical cross-linking to finely tune the dielectric constant and optimise interfacial impedance matching has been elucidated. Furthermore, the flexibility and tunability of PVA endow the shielding materials with multifunctional properties that integrate thermal conductivity, flame retardancy, smart sensing, self-healing and phase-change energy storage, demonstrating immense application potential in the fields of flexible wearable protective electronics and lightweight, high-energy protection for the aerospace sector.

Although PVA offers advantages such as excellent film-forming properties, an abundance of hydroxyl groups, strong hydrogen-bonding capacity, processability, flexibility and biocompatibility, its inherent hydrophilicity poses significant challenges for PVA-based EMI shielding materials in practical applications. Whilst this hydrophilicity is beneficial for the preparation of hydrogels and flexible composites, it is a double-edged sword in complex application scenarios. Under high-humidity conditions, the PVA matrix readily absorbs water and undergoes severe swelling, resulting in poor wet-heat stability.^118,124^ This volumetric expansion increases the spatial distance between adjacent fillers, disrupting the carefully constructed continuous conductive network and thereby causing a sharp decline in EMI shielding performance. Furthermore, in MXene/PVA composite systems, absorbed water molecules form a water-rich microenvironment within the matrix, which accelerates the degradation of highly reactive MXene nanosheets into non-conductive oxides, thereby permanently impairing their intrinsic electrical conductivity.^125^

Apart from the moisture absorption issues associated with solid-state composites, PVA-based hydrogel shielding materials also face significant environmental adaptability challenges. In open, dry environments, hydrogels are prone to water evaporation (dehydration), leading to macroscopic structural shrinkage, hardening and embrittlement; at sub-zero temperatures, the freezing of large amounts of water within the PVA network causes them to completely lose their flexibility and structural integrity.^126,127^ To overcome these limitations, future research should focus on developing the following strategies: constructing robust chemically cross-linked networks, designing hydrophobic protective surface layers, and introducing anti-drying/anti-freezing binary solvent systems (such as water/glycerol mixtures),^128,129^ to ensure the long-term reliability of PVA-based shielding materials in complex operational environments.

## Author contributions

Rui Zheng: conceptualisation, data curation, writing – original draft, writing – review & editing. Jiang Yang: data curation, supervision, investigation. Zihuan Weng: data curation, supervision, investigation. Yuzhu Xiong: supervision, funding acquisition, writing – review & editing.

## Conflicts of interest

The authors declare that they have no conflicts of interest.

## Data Availability

No primary research results, software or code have been included and no new data were generated or analyzed as part of this review.
